# The risk of dementia in breast cancer survivors: a meta-analysis of observational studies

**DOI:** 10.1080/07853890.2025.2529579

**Published:** 2025-07-16

**Authors:** Shijun Tan, Jiawei Yang, Guiping Guo, Shicui Hong, Li Guo, Honglin Situ

**Affiliations:** a Guangzhou University of Chinese Medicine, Guangzhou, Guangdong, China; b Department of Mammary Disease, Guangdong Provincial Hospital of Chinese Medicine, Guangzhou, Guangdong, China

**Keywords:** Breast cancer, dementia, cognitive disorders, meta-analysis

## Abstract

**Objective:**

Cognitive problems are among the most common post-treatment symptoms experienced by breast cancer patients, raising concerns about their long-term cognitive health. This meta-analysis aims to clarify the relationship between cognitive decline in breast cancer survivors and the risk of developing dementia.

**Materials and methods:**

PubMed, Embase, and Cochrane Library were searched for cohort studies published from database inception to August 27, 2024, using medical subject headings (MeSH) and keywords. All statistical analyses were performed using Stata statistical software version 14.0. If *p* > 0.1 and I^2^ ≤50%, a fixed-effects model was adopted. If I^2^ > 50%, a random-effects model was adopted. The funnel plot and Egger’s test were used to evaluate publication bias.

**Results:**

The meta-analysis, comprising 9 studies and involving 887,678 individuals, revealed that breast cancer survivors did not exhibit an increased risk of all-cause dementia [RR = 0.997, 95%CI (0.965, 1.029); I^2^=0.0%, *p* = 0.386], Intriguingly, endocrine therapy [RR = 0.904, 95%CI (0.865, 0.946); I^2^ = 41.7%, *p* = 0.161] and chemotherapy [RR = 0.754, 95%CI (0.604, 0.940); I^2^ = 0.0%, *p* = 0.592] may even serve as potential protective factors against dementia in breast cancer survivors.

**Conclusion:**

This meta-analysis indicates that breast cancer survivors do not face an elevated risk of all-cause dementia. Furthermore, treatments such as endocrine therapy and chemotherapy may have a protective effect against dementia. Further research is needed to explore the underlying mechanisms and confirm these findings.

## KEY MESSAGES

Breast cancer survivors do not face an elevated risk of all-cause dementia. Endocrine therapy and chemotherapy may have a protective effect against dementia.

## Introduction

Breast cancer is the most commonly diagnosed cancer among women, as reported by Global Cancer Statistics 2020 [1]. A 2025 study in Nature Medicine analyzing GLOBOCAN 2022 data revealed 2.3 million new global cases that year, with half of surveyed countries experiencing annual incidence increases of 1–5% [2]. Advances in screening and treatment modalities – including surgery, radiation, chemotherapy, endocrine therapy (ET), and targeted therapies – have significantly reduced breast cancer mortality and improved 5-year survival rates, which now exceed 90% in high-resource settings [3]. However, this progress raises concerns about the quality of life for survivors and has shifted research focus toward long-term survivorship issues. While cardiovascular diseases (e.g. myocardial injury, heart failure), metabolic disorders (e.g. diabetes, dyslipidemia), and osteoporosis in survivors have been extensively studied [4,5], cognitive impairment – particularly regarding cognitive issues, which are among the most frequent post-treatment symptoms [6] – remains underrecognized by both patients and clinicians. Cognitive problems, affecting memory and thinking, are especially prevalent in older adults aged 65 and above [7], and the Global Burden of Disease estimates that 57.4 million people lived with dementia in 2019, a number expected to nearly triple to 152.8 million by 2050 [8].

Research indicates that cognitive decline may occur following breast cancer treatment, particularly related to ET and chemotherapy. ET, the standard care for hormone receptor-positive breast cancers, is selected based on factors such as menopausal status and tumor characteristics, with common medications including aromatase inhibitors (AIs) and selective estrogen receptor modulators (SERMs) [9]. Studies have shown that breast cancer patients undergoing ET may experience negative effects on cognitive function, including lower verbal memory scores compared to those without systemic therapy [10–13]. Similarly, chemotherapy, while effective in reducing recurrence rates, can induce neurotoxicity and lead to chemotherapy-induced cognitive impairment, affecting a significant percentage of cancer survivors [14–16].

Despite the evidence of cognitive decline, the relationship between breast cancer and the risk of developing dementia remains unclear, with some studies indicating a higher risk while others find no significant association [17–19]. Given the conflicting results, we propose the hypothesis and clinical questions: Is the risk of cognitive impairment higher for breast cancer survivors? To address these critical issues, our meta-analysis aims to systematically evaluate the risk of dementia among breast cancer survivors in comparison to the general population and to assess the long-term cognitive effects of cancer treatments. The findings will provide vital evidence to guide clinical management and survivorship care strategies.

## Method

Our meta-analysis adheres to the latest standards and guidelines for systematic reviews and meta-analysis [20]. The protocol for this study has been registered with PROSPERO, under registration number CRD42023458100.

### Data sources

A comprehensive literature search was conducted from the inception of the databases until August 27, 2024, utilizing EMBASE, PubMed, and the Cochrane Library, with no restrictions applied. We employed subject terms and relevant keywords related to cognitive dysfunction, dementia, and breast neoplasm, including their variants. Additionally, we reviewed the citation lists of the retrieved studies and previous meta-analysis to identify additional relevant studies for inclusion. Detailed search strategies for these databases are provided in Supplementary Tables 1–3.

**Table 1.** Basic characteristics of the included studies.

References	Country	Study type	Sample size (exposure/control group)	Age in exposure/control group	Race	Control group	No. of outcome	Type of dementia	Follow-up period (exposure/control group)	Diagnosis	Confounders adjusted
***Healthy controls***											
Wennberg (2023)	Sweden	Cohort study	26,741 249,540	60.1 ± 5.9 60.4 ± 6.0	/	Healthy people	2,847	ACD AD VaD	8.8 ± 4.5 years	ICD-9 codes	Age, education, country of origin
Carreira (2021)	UK	Cohort study	57,571 230,067	/	White South Asian Black Other and mixed Unknown	Healthy people	24,213	ACD	Median 4.5 years 5.2 years	ICD-9 codes	Age, primary care practice, diabetes, BMI, smoking, drinking status
Sun (2016)	Taiwan	Cohort study	96,788 24,197	49.30 ± 0.0949.49 ± 0.04	/	Healthy people	527	ACD	7.4 ± 3.0 years 8.4 ± 2.1 years	ICD-9 codes	Age, comorbidity, surgery, chemotherapy, radiotherapy, aromatase inhibitor therapy, benzodiazepine
***No ET controls***											
Chao Cai (2024)	USA	Cohort study	12,356 6,452	75 76	White Black Other	Breast cancer patients without ET	4728	AD	>10 years	ICD-9/ICD-10 codes	Age, race, marital status, nursing home resident, poverty indicator, medical factors, treatment factors
Thompso (2021)	USA	Cohort study	1,9128 6,649	73.9 ± 6.2 76.0 ± 7.4	White Black Other	Breast cancer patients without ET	2,869	ACD AD VaD	Median 2.7 years	ICD-9 codes	Age, race, region, marital status, TN stage, Charlson Comorbidity Index, treatment with radiation/surgery/chemotherapy
Branigan (2020)	USA	Cohort study	18,126 39,717	76.2 ± 7.0 76.8 ± 7.0	White Black Asian Hispanic North American Native Other Unknown	Breast cancer patients without ET	8,042	ACD AD	5.5 ± 1.8 years	ICD-9/ICD-10 codes	Age, race/ethnicity, comorbidities, Charlson Comorbidity Index
Sun (2016)	Taiwan	Cohort study	96,788 24,197	49.30 ± 0.09 49.49 ± 0.04	/	Breast cancer patients without using TAM	527	ACD	7.4 ± 3.0 years 8.4 ± 2.1 years	ICD-9 codes	Age, comorbidity, surgery, chemotherapy, radiotherapy, aromatase inhibitor therapy, benzodiazepine
Ording (2013)	Denmark	Cohort study	6,141 10,278	/	/	Breast cancer patients without ET	501	ACD AD VaD	/	ICD-9 codes	Menopausal status, calendar period of breast cancer diagnosis
***No chemotherapy controls***											
Ording (2013)	Denmark	Cohort study	3,389 11,030	/	/	Breast cancer patients without chemotherapy	501	ACD	/	ICD-9 codes	Menopausal status, calendar period of breast cancer diagnosis
Du (2010)	USA	Cohort study	14,057 48,508	72(65-89) 72(65-89)	White African-American Others	Breast cancer patients without chemotherapy	/	ACD AD VaD	Median 16.0 years	ICD-9 codes	Age, ethnicity, marital status, tumor stage, tumor grade, tumor size, hormone receptor status, comorbidity, radiation therapy, socioeconomic status, year of diagnosis, SEER areas.
Baxter (2009)	USA	Cohort study	2,913 18,449	70 73	Non-African American African American	Breast cancer patients without chemotherapy	772	ACD	Median 4.9 years	ICD-9 codes	Age, ethnicity, marital status, tumor stage, tumor grade, tumor size, hormone receptor status, comorbidity, radiation therapy, socioeconomic status, year of diagnosis, SEER areas

*ICD: International Classification of Diseases; ACD: all-cause dementia; ET : endocrinotherapy; TAM: tamoxifen; SEER: Surveillance, Epidemiology, and End Results.

**Table 2.** The quality assessment of cohort studies.

Study	Year	Selection	Comparability	Outcome	Total
Cohort studies (*n* = 9)					
Chao Cai et al.	2024	****	**	***	9
Wennberg et al.	2023	****	**	***	9
Thompson et al.	2021	****	**	**	8
CarreiraI et al.	2021	****	**	**	8
Branigan et al.	2020	****	*	**	7
Sun et al.	2016	****	**	**	8
Ording et al.	2013	****	/	**	6
Du et al.	2010	****	**	**	8
Baxter et al.	2009	****	**	**	8

**Table 3.** Subgroup analysis for the risk of dementia in receiving different ET medicine and different type’s dementia risk in patients undergoing ET.

Subgroups	Included studies	RR (95% CI)	Heterogeneity	
			I^2^ (%)	*P*-values
Medicine type				
SERMs	5	0.920 (0.822–1.029)	81.3%	0.000
AIs	3	0.899 (0.833–0.970)	67.2%	0.047
Dementia type				
AD	4	0.898 (0.811–0.994)	66.0%	0.032
VaD	2	0.998 (0.824–1.208)	26.5%	0.243

### Study selection

Initially, all retrieved records were imported into the reference management software EndNote, where duplicate entries were removed. Subsequently, two authors (SJ Tan and GP Guo) independently evaluated the titles and abstracts to eliminate irrelevant records. The remaining studies were classified as inclusion, exclusion, or uncertain. For those categorized as uncertain, a full-text review was conducted to determine eligibility. Any disagreements between the authors were resolved through group discussions.

### Eligibility criteria

Studies were deemed eligible based on the following criteria: (a) inclusion of individuals diagnosed with dementia, vascular dementia (VaD), or Alzheimer’s disease (AD); (b) confirmed diagnosis of breast cancer and receipt of standard treatment; (c) comparison groups consisting of healthy individuals, non-cancer patients, or breast cancer patients receiving non-targeted treatments; (d) reported relative risk (RR), hazard ratio (HR), or odds ratio (OR) with associated confidence intervals (CIs) indicating the association between dementia, AD, or VaD risk in breast cancer survivors, along with adjustments for potential confounders; and (e) inclusion of cohort studies. Exclusion criteria included: (a) duplicate publications, (b) editorials or conference abstracts, and (c) incomplete data or absence of relevant outcomes.

### Data extraction

A data extraction table was developed using Microsoft Excel. Both authors, SJ Tan and JW Yang, independently extracted data from the eligible cohorts. The extracted information included details such as the country of study, first author, publication date, number of events and exposures, and confounders. The extracted data were cross-checked for accuracy, and any discrepancies were resolved through discussion.

### Study quality

The quality of the included studies was assessed using the Newcastle-Ottawa Quality Assessment Scale [21] focusing on three key areas: selection, comparability, and outcomes. Case-control and cohort studies were scored on a scale from 0 to 9, with higher scores indicating higher quality. Studies with scores of ≥7 were classified as high quality, those scoring between 4 and 6 were considered moderate quality, while scores ranging from 0 to 3 were categorized as low quality.

### Data synthesis

Data analysis was performed using Stata software (version 14). Heterogeneity among studies was evaluated using the chi-square test and I^2^ statistic, where a significance level of *p* < 0.1 or I^2^>50% indicated substantial heterogeneity, prompting the use of a random-effects model. Conversely, a fixed-effect model was applied when these criteria were not met [22]. To assess the robustness of the overall findings and explore potential sources of heterogeneity, sensitivity analyses were conducted. Subgroup analyses were performed based on specific endocrine therapy agents and types of dementia. Finally, potential publication bias was evaluated using Egger’s test and funnel plots [23].

## Result

### Study selection

We generated 3142 relevant items, and 553 were removed because of duplication. Subsequently, 2566 records were eliminated through the screening of titles and abstracts as they were found to be unrelated to our subject. The final 23 studies underwent further scrutiny for evaluation. Ultimately, 9 studies [17,19, 24–30] in all satisfied the requirements for inclusion in the meta-analysis.

**Figure 1.**
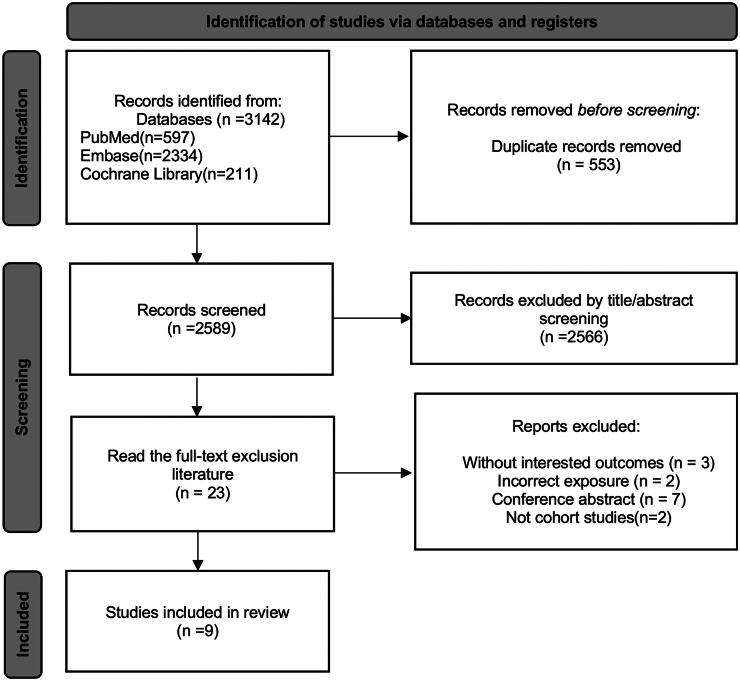
Literature screening flowchart.

Figure 1 shows the study selection procedure.

### Study characteristics

A comprehensive analysis involved nine cohort studies, encompassing a total of 887,678 individuals, among whom 44,505 experienced dementia events. These studies spanned publication years from 2009 to 2024, with sample sizes ranging from 16,419 to 287,638. The follow-up periods varied from 2 to 16 years. Among these studies, five studies were conducted in the United States [24–26,29,30], and the remaining four were carried out in Sweden [17], the United Kingdom [19], Denmark [28], and Taiwan [27], respectively. The primary method for diagnosing dementia in the original studies was predominantly according to the criteria outlined in the ‘International Classification of Diseases (ICD)’. The adjusted confounders in each study differed marginally, with age, race, and comorbidities being the most adjusted factors. A summary detailing the features of the 9 research is shown in Table 1, and specific data for the dataset can be found in Supplementary Tables 4–7.

### Quality assessment

Based on the NOS criteria, the average score for all included studies was 7.8. This implies that the research included in this meta-analysis is of a high caliber. Further details regarding the scores of the included studies are shown in Table 2.

### Breast cancer survivor and risk of dementia

**Figure 2.**
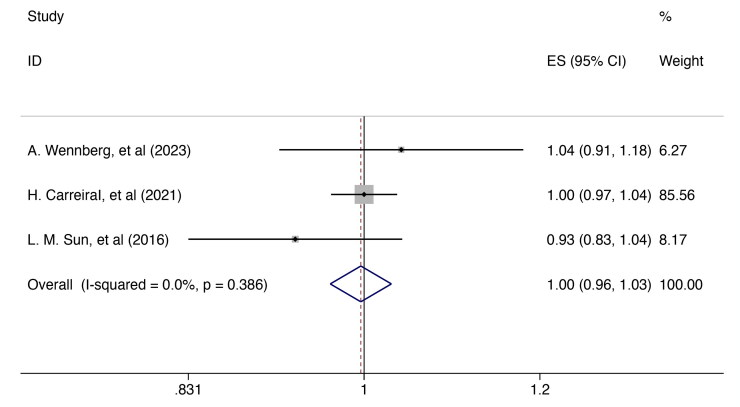
Forest plot for the risk of all-cause dementia in breast cancer survivor. ES: effect size.

Three cohorts [17,19,27] offered information on breast cancer patients’ risk of all-cause dementia compared with healthy people. According to the pooled statistics, the incidence of all-cause dementia was not significantly correlated with breast cancer survivors [RR = 0.997, 95%CI (0.965, 1.029); I^2^=0.0%, *p* = 0.386; Figure 2]. None of the single studies had a significant impact on reversing the pooled effect in the sensitivity analysis, suggesting that the findings on dementia risk in breast cancer survivors are robust (Supplementary Figure 1).

### Breast cancer survivors received ET and risk of dementia

**Figure 3.**
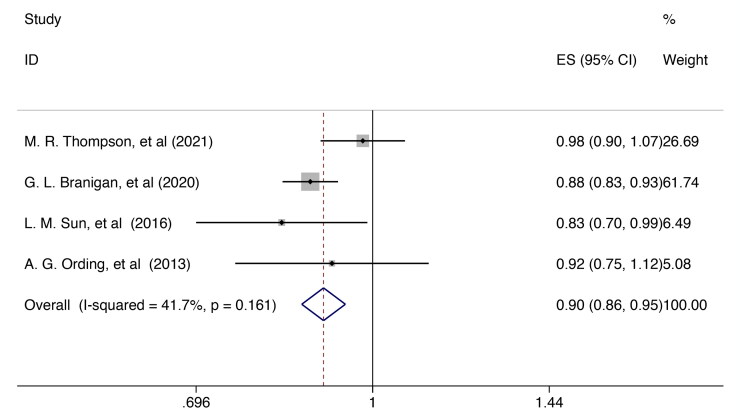
Forest plot for the risk of dementia in breast cancer survivor received ET. ES, effect size.

Four research [25–28] were included in the assessment of the relationship between breast cancer survivor received ET and all-cause dementia risk. The findings support the notion that endocrine therapy may serve as a potential protective factor against all-cause dementia [RR = 0.904, 95%CI (0.865, 0.946); I^2^ = 41.7%, *p* = 0.161; Figure 3]. Given the moderate heterogeneity observed, we conducted a sensitivity analysis through systematically eliminated each study to determine the possible cause of heterogeneity. The results of this analysis indicated stability, as the general conclusion remained largely unchanged when any one study was excluded (Supplementary Figure 2).

### Breast cancer survivor received chemotherapy and risk of dementia

**Figure 4.**
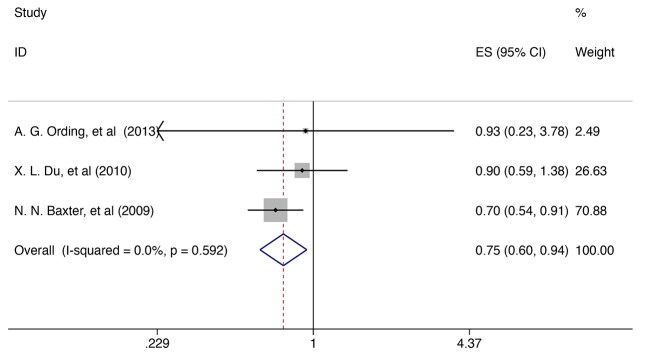
Forest plot for the risk of dementia in breast cancer survivor received chemotherapy. ES: effect size.

Three research [28–30] were included in investigating the correlation between breast cancer patients undergoing chemotherapy and the risk of all-cause dementia, revealing that patients who underwent chemotherapy had a reduced risk of dementia [RR = 0.754, 95% CI (0.604, 0.940); I^2^ = 0.0%, *p* = 0.592; Figure 4]. None of the single studies had a significant impact on reversing the pooled effect in the sensitivity analysis, suggesting that the findings are robust (Supplementary Figure 3).

### Subgroup analysis

We conducted a subgroup analysis to examine the effects of different ET medications on the risk of all-cause dementia in breast cancer patients. Among the studies reviewed, five [24–28] provided data on the impact of SERMs on dementia risk. The results indicated that SERMs did not significantly influence the risk of dementia, with an RR of 0.920 (95% CI: 0.822, 1.029), demonstrating high heterogeneity (I^2^ = 81.3%, *p* = 0.000; Table 3).

In contrast, three studies [24–26] investigated the association between AIs and the risk of all-cause dementia, revealing that patients taking AIs exhibited a reduced risk of dementia, with an RR of 0.899 (95% CI: 0.833, 0.970), and moderate heterogeneity (I^2^ = 67.2%, *p* = 0.047; Table 3).

Additionally, we performed a subgroup analysis based on the types of dementia. Due to limited data available in the literature, only breast cancer patients undergoing ET were included in this analysis. The findings suggested that ET provided protective effects against the onset of AD [24–26,28] but did not have a significant impact on VaD [25,28]. Specifically, the RR for AD was 0.898 (95% CI: 0.811, 0.994), with moderate heterogeneity (I^2^ = 66.0%, *p* = 0.032), while the RR for VaD was 0.998 (95% CI: 0.824, 1.208), indicating low heterogeneity (I^2^ = 26.5%, *p* = 0.243; Table 3).

### Publication bias

**Figure 5.**
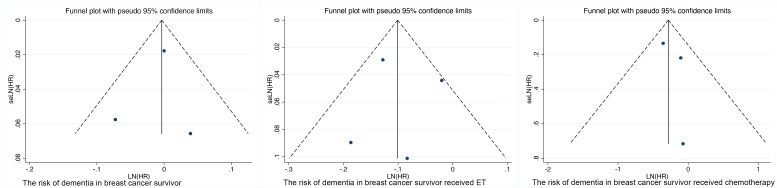
Funnel plot for all-cause dementia in breast cancer survivor.

A visual inspection of the funnel plot indicates no significant evidence of publication bias concerning the association between the risk of all-cause dementia and breast cancer survivors. This finding is further corroborated by the results of Egger’s test, which yielded a *P*-value of 0.821, suggesting no substantial bias. Moreover, the funnel plot analysis examining the effects of ET and chemotherapy on cognitive performance also revealed no discernible publication bias. In Egger’s test, the *P*-values were 0.952 for ET and 0.555 for chemotherapy, both indicating an absence of publication bias. The funnel plot is illustrated in Figure 5.

## Discussion

### Main findings

This meta-analysis included nine studies with a total of 887,678 participants, providing a comprehensive assessment of the relationship between breast cancer survivors and dementia. Our findings did not support a significant link between breast cancer and the risk of dementia. Notably, we observed a negative correlation between ET, chemotherapy, and dementia risk, despite the known detrimental effects of cancer treatments on cognitive function.

### Interpretation of findings

In reviewing prior research, a meta-analysis of seven studies confirmed a negative association between the risk of AD and a history of cancer [31]. However, this analysis lacked consideration for cancer types and the cognitive effects of cancer treatment. A more recent review explored the connection between cancer and dementia, revealing a lower risk of dementia among cancer survivors [32]. Within this review, five retrospective cohort studies on breast cancer survivors demonstrated a significant negative association with AD. Subgroup analysis indicated that breast cancer survivors undergoing ET or chemotherapy had a reduced risk of AD. Nonetheless, this meta-analysis, focused on breast cancer, relied on only five studies, potentially limiting the objectivity and accuracy of its conclusions. In contrast, our current analysis incorporates more recent studies, examining data across various dementia types and treatments, aiming to substantiate robust evidence for the link between breast cancer survivors and dementia.

The precise relationship between cancer and dementia remains complex and not fully understood, yet a substantial body of literature suggests a negative correlation between the two [33–36]. Several plausible pathophysiological mechanisms may explain this connection. Cancer typically activates cell proliferation and survival pathways, while dementia is associated with pathways leading to cell death [37,38]. For instance, the tumor suppressor protein p53 is often reduced in cancer but elevated in the brains of dementia patients. Similarly, the peptidylprolyl cistrans isomerase, essential for cell cycle regulation and protein folding, is generally overactivated in tumors but depleted in dementia. Additionally, contrasting disturbances in epigenetic homeostasis may contribute to the slower development of AD in cancer patients, including an increased tendency toward aberrant hypermethylation that may affect the promoters of beta-amyloid processing enzymes, thereby reducing the formation of amyloid plaques [39].

Despite these findings and potential biological mechanisms, the observational studies included in this meta-analysis have inherent methodological limitations, including monitoring bias and survival bias [37]. Therefore, it is crucial to critically evaluate the results, raising questions about the existence of a true inverse relationship between dementia and cancer. Some evidence suggests a positive relationship between the two. Cancer can alter normal brain function through mechanisms such as immune inflammatory responses, oxidative stress, and angiogenesis, which may jointly influence the development of both cancer and dementia. The immune response triggered by cancer can mediate central nervous system inflammation [40–42]. The tumor suppressor protein BRCA1, significant in breast cancer, is linked to AD, with its dysregulation promoting neuronal cell death [43]. Emotional issues such as anxiety, depression, and fatigue experienced by cancer patients [44], along with the effects of surgical anesthesia [45], may also negatively impact cognitive function. Additionally, common risk factors such as physical inactivity, poor diet, and aging can contribute to the development of both conditions [37]. In summary, our data indicate no correlation between dementia risk and breast cancer survivors, leaving the underlying mechanisms for future exploration. The interconnected pathways between cancer and dementia form a complex relationship.

Our analysis also explored the connection between breast cancer treatment and dementia. Patients with a history of ET or chemotherapy exhibited a slightly lower risk of dementia compared to those who did not receive these treatments. Notably, AIs emerged as a protective factor for cognition in subgroup analyses, while SERMs did not show a similar effect. The two classes of medications operate through different mechanisms, with circulating estrogen levels significantly lower following AI treatment than with SERMs. Given the cognitive protective effects of estrogen [46], one might expect AIs to have a more detrimental impact on cognition. However, our findings suggest the opposite, supported by existing studies. Randomized controlled trials have shown that patients on letrozole, an AI, demonstrated better overall cognitive performance than those on tamoxifen, a classic SERM [47]. Exemestane, another AI, has metabolites with mild androgenic properties that may protect cognition [48]. Although some studies indicate cognitive decline in breast cancer patients receiving ET [13,47,49], a subsequent study found significant cognitive improvement one year after completing treatment, suggesting the potential reversibility of ET’s negative effects [50]. Thus, regardless of the specific ET medication used, ET may not increase dementia risk in breast cancer patients and could even serve as a protective factor.

Regarding chemotherapy, some cancer patients undergoing specific treatments report cognitive impairment, commonly referred to as ‘chemo-brain’ [16]. It has been linked to challenges in decision-making and daily functioning, and some studies suggest cognitive-behavioral therapy and tailored psychological interventions may help [51]. Less clear, however, is whether chemotherapy -related cognitive changes influence dementia risk. One study identified reductions in frontal gray matter and associated executive dysfunction in breast cancer patients post-chemotherapy [52]. Yet, a meta-analysis evaluating cognitive performance in breast cancer patients who completed chemotherapy over six months found only minimal and localized residual cognitive impairment in verbal and visuospatial functions [53]. This reversible change is supported by functional magnetic resonance imaging, which shows a decrease in brain area density after chemotherapy, followed by an increase after one year. Additionally, replacement brain areas exhibited higher activation, suggesting the initiation of compensatory mechanisms for brain function [54]. On a pathophysiological level, taxanes, commonly used in breast cancer chemotherapy regimens, may have neuroprotective effects by stabilizing microtubules and are proposed as potential therapeutic agents for AD [55]. Consequently, chemotherapy may exert a protective influence on long-term cognitive function in breast cancer patients, potentially lowering the risk of dementia.

### Implications and limitations

Our meta-analysis examines the correlation between breast cancer survivorship and the risk of dementia, revealing that neither breast cancer nor its treatments increase dementia risk. In fact, ET and chemotherapy may act as potential protective factors. This research offers potential relief from the psychological burden for some breast cancer patients. While breast cancer and its treatments may temporarily impact cognitive function post-treatment, these effects appear limited and reversible, with no lasting impact on cognitive status or quality of life for breast cancer patients. However, our study has limitations. Firstly, it exclusively includes retrospective studies, so future research should incorporate prospective studies for a more comprehensive investigation. Additionally, the analysis poses challenges due to the majority of patients undergoing multimodal treatment, making it difficult to isolate the impact of each component on cognition. Lastly, the younger age at which breast cancer is diagnosed compared to other cancers may result in insufficient follow-up time in the literature, potentially overlooking the onset of dementia outcomes.

## Conclusion

This meta-analysis indicates that breast cancer survivors do not face an elevated risk of all-cause dementia. Moreover, ET and chemotherapy may serve as potential protective factors against dementia. However, further studies are necessary to validate the underlying pathophysiological mechanisms driving this phenomenon.

## Supplementary Material

Supplementary Material.docx

## Data Availability

The datasets presented in this study can be found in the supplementary material.
