# Supplementary material for: The risk of dementia in breast cancer survivors: a meta-analysis of observational studies
Source: Ann Med. 2025 Jul 16;57(1):2529579. doi: 10.1080/07853890.2025.2529579 (PMC12269053; doi:10.1080/07853890.2025.2529579)
Supplement: Supplementary Material.docx [file IANN_A_2529579_SM2972.docx]

Supplementary Material

# Supplementary Tables

**Table 1~3: Details of the Literature Search Strategy**

**(1) PubMed (****August 27, 2024)**

| **Search** | **Query** | **Items found** |
| --- | --- | --- |
| #1 | "Cognitive Dysfunction"[MeSH Terms] OR "Dementia"[MeSH Terms] OR "Alzheimer Disease"[MeSH Terms] | 245,021 |
| #2 | "cognitive dysfunction*"[Title/Abstract] OR "cognitive impairment*"[Title/Abstract] OR "cognitive disorder*"[Title/Abstract] OR "cognitive impairment*"[Title/Abstract] OR "cognitive decline*"[Title/Abstract] OR "mental deterioration*"[Title/Abstract] OR "Dementia"[Title/Abstract] OR "amentia*"[Title/Abstract] OR "alzheimer disease"[Title/Abstract] OR "alzheimer dementia*"[Title/Abstract] OR "alzheimer s disease*"[Title/Abstract] OR "alzheimer sclerosis"[Title/Abstract] OR "alzheimer syndrome"[Title/Abstract] | 375,262 |
| #3 | "Cognitive Dysfunction"[MeSH Terms] OR "Dementia"[MeSH Terms] OR "Alzheimer Disease"[MeSH Terms] OR "cognitive dysfunction*"[Title/Abstract] OR "cognitive impairment*"[Title/Abstract] OR "cognitive disorder*"[Title/Abstract] OR "cognitive impairment*"[Title/Abstract] OR "cognitive decline*"[Title/Abstract] OR "mental deterioration*"[Title/Abstract] OR "Dementia"[Title/Abstract] OR "amentia*"[Title/Abstract] OR "Alzheimer Disease"[Title/Abstract] OR "alzheimer dementia*"[Title/Abstract] OR "alzheimer s disease*"[Title/Abstract] OR "alzheimer sclerosis"[Title/Abstract] OR "alzheimer syndrome"[Title/Abstract] | 423,822 |
| #4 | "Breast Neoplasms"[MeSH Terms] | 357,463 |
| #5 | "breast neoplasm*"[Title/Abstract] OR "breast tumor*"[Title/Abstract] OR "breast cancer*"[Title/Abstract] OR "mammary cancer*"[Title/Abstract] OR "breast malignant tumor*"[Title/Abstract] OR "mammary carcinoma*"[Title/Abstract] OR "mammary neoplasm*"[Title/Abstract] OR "breast carcinoma*"[Title/Abstract] | 404,327 |
| #6 | "Breast Neoplasms"[MeSH Terms] OR "breast neoplasm*"[Title/Abstract] OR "breast tumor*"[Title/Abstract] OR "breast cancer*"[Title/Abstract] OR "mammary cancer*"[Title/Abstract] OR "breast malignant tumor*"[Title/Abstract] OR "mammary carcinoma*"[Title/Abstract] OR "mammary neoplasm*"[Title/Abstract] OR "breast carcinoma*"[Title/Abstract] | 484,404 |
| #7 | ("Breast Neoplasms"[MeSH Terms] OR ("breast neoplasm*"[Title/Abstract] OR "breast tumor*"[Title/Abstract] OR "breast cancer*"[Title/Abstract] OR "mammary cancer*"[Title/Abstract] OR "breast malignant tumor*"[Title/Abstract] OR "mammary carcinoma*"[Title/Abstract] OR "mammary neoplasm*"[Title/Abstract] OR "breast carcinoma*"[Title/Abstract])) AND ("Cognitive Dysfunction"[MeSH Terms] OR "Dementia"[MeSH Terms] OR "Alzheimer Disease"[MeSH Terms] OR ("cognitive dysfunction*"[Title/Abstract] OR "cognitive impairment*"[Title/Abstract] OR "cognitive disorder*"[Title/Abstract] OR "cognitive impairment*"[Title/Abstract] OR "cognitive decline*"[Title/Abstract] OR "mental deterioration*"[Title/Abstract] OR "Dementia"[Title/Abstract] OR "amentia*"[Title/Abstract] OR "Alzheimer Disease"[Title/Abstract] OR "alzheimer dementia*"[Title/Abstract] OR "alzheimer s disease*"[Title/Abstract] OR "alzheimer sclerosis"[Title/Abstract] OR "alzheimer syndrome"[Title/Abstract])) | 1,974 |
| #8 | "Risk"[MeSH Terms] | 1,429,175 |
| #9 | "Risk"[Title/Abstract] OR "Risk"[MeSH Terms] | 3,504,478 |
| #10 | ("Risk"[Title/Abstract] OR "Risk"[MeSH Terms]) AND (("Breast Neoplasms"[MeSH Terms] OR ("breast neoplasm*"[Title/Abstract] OR "breast tumor*"[Title/Abstract] OR "breast cancer*"[Title/Abstract] OR "mammary cancer*"[Title/Abstract] OR "breast malignant tumor*"[Title/Abstract] OR "mammary carcinoma*"[Title/Abstract] OR "mammary neoplasm*"[Title/Abstract] OR "breast carcinoma*"[Title/Abstract])) AND ("Cognitive Dysfunction"[MeSH Terms] OR "Dementia"[MeSH Terms] OR "Alzheimer Disease"[MeSH Terms] OR ("cognitive dysfunction*"[Title/Abstract] OR "cognitive impairment*"[Title/Abstract] OR "cognitive disorder*"[Title/Abstract] OR "cognitive impairment*"[Title/Abstract] OR "cognitive decline*"[Title/Abstract] OR "mental deterioration*"[Title/Abstract] OR "Dementia"[Title/Abstract] OR "amentia*"[Title/Abstract] OR "Alzheimer Disease"[Title/Abstract] OR "alzheimer dementia*"[Title/Abstract] OR "alzheimer s disease*"[Title/Abstract] OR "alzheimer sclerosis"[Title/Abstract] OR "alzheimer syndrome"[Title/Abstract]))) | 597 |

**(2)** **Cochrane Library (August 27, 2024)**

| **Search** | **Query** | **Items found** |
| --- | --- | --- |
| #1 | MeSH descriptor: [Cognitive Dysfunction] explode all trees | 4093 |
| #2 | MeSH descriptor: [Dementia] explode all trees | 9541 |
| #3 | MeSH descriptor: [Alzheimer Disease] explode all trees | 5391 |
| #4 | (Cognitive Dysfunction*):ti,ab,kw OR (Cognitive Impairment*):ti,ab,kw OR (Cognitive Disorder*):ti,ab,kw OR (Cognitive Impairment*):ti,ab,kw OR (Cognitive Decline*):ti,ab,kw OR(Mental Deterioration*):ti,ab,kw OR (Dementia):ti,ab,kw OR (Amentia*):ti,ab,kw OR (Alzheimer Disease):ti,ab,kw OR (Alzheimer Dementia*):ti,ab,kw OR (Alzheimer Sclerosis):ti,ab,kw OR (Alzheimer Syndrome):ti,ab,kw (Word variations have been searched) | 78575 |
| #5 | #1 OR #2 OR #3 OR #4 | 78898 |
| #6 | MeSH descriptor: [Breast Neoplasms] explode all trees | 20356 |
| #7 | (Breast Neoplasm*):ti,ab,kw OR (Breast Tumor*):ti,ab,kw OR (Breast Cancer*):ti,ab,kw OR (Mammary Cancer*):ti,ab,kw OR (Breast Malignant Tumor*):ti,ab,kw OR (Mammary Carcinoma*):ti,ab,kw OR (Mammary Neoplasm*):ti,ab,kw OR (Breast Carcinoma*):ti,ab,kw (Word variations have been searched) | 48948 |
| #8 | #6 OR #7 | 48948 |
| #9 | #5 AND #8 | 906 |
| #10 | MeSH descriptor: [Risk] explode all trees | 56620 |
| #11 | (Risk):ti,ab,kw (Word variations have been searched) | 320185 |
| #12 | #10 OR #11 | 324098 |
| #13 | #12 AND #9 | 211 |

**(3) Embase (August 27, 2024)**

| **Search** | **Query** | **Items found** |
| --- | --- | --- |
| #1 | 'cognitive defect'/exp OR 'dementia'/exp OR 'alzheimer disease'/exp | 661,909 |
| #2 | 'cognitive dysfunction*':ti,ab,kw OR 'cognitive disorder*':ti,ab,kw OR 'cognitive impairment*':ti,ab,kw OR 'cognitive decline*':ti,ab,kw OR 'mental deterioration*':ti,ab,kw OR dementia:ti,ab,kw OR amentia*:ti,ab,kw OR 'alzheimer disease':ti,ab,kw OR 'alzheimer dementia*':ti,ab,kw OR 'alzheimers disease*':ti,ab,kw OR 'alzheimer sclerosis':ti,ab,kw OR 'alzheimer syndrome':ti,ab,kw | 396,236 |
| #3 | #1 OR #2 | 725,812 |
| #4 | 'breast tumor'/exp | 719,369 |
| #5 | 'breast neoplasm*':ti,ab,kw OR 'breast tumor*':ti,ab,kw OR 'breast cancer*':ti,ab,kw OR 'mammary cancer*':ti,ab,kw OR 'breast malignant tumor*':ti,ab,kw OR 'mammary carcinoma*':ti,ab,kw OR 'mammary neoplasm*':ti,ab,kw OR 'breast carcinoma*':ti,ab,kw | 585,011 |
| #6 | #4 OR #5 | 785,337 |
| #7 | #3 AND #6 | 7,672 |
| #8 | 'risk'/exp | 3,283,363 |
| #9 | risk:ti,ab,kw | 4,316,074 |
| #10 | #8 OR #9 | 5,261,920 |
| #11 | #7 AND #10 | 2,334 |

**Table 4~7: Details of the Datasets**

**(4) Dataset of** **all-cause dementia and breast cancer survivors (healthy controls)**

| **Author** | **Year** | **HR/OR** | **LL** | **UL** |
| --- | --- | --- | --- | --- |
| A. Wennberg | 2023 | 1.04 | 0.92 | 1.19 |
| H. CarreiraI | 2021 | 1.00 | 0.97 | 1.04 |
| L. M. Sun | 2016 | 0.93 | 0.83 | 1.04 |

**(5)** **Dataset of all-cause dementia and breast cancer survivors received ET (No ET controls)**

| **Author** | **Year** | **HR/OR** | **LL** | **UL** |
| --- | --- | --- | --- | --- |
| M. R. Thompson, et al | 2021 | 0.98 | 0.9 | 1.07 |
| G. L. Branigan, et al | 2020 | 0.88 | 0.83 | 0.93 |
| L. M. Sun, et al | 2016 | 0.83 | 0.69 | 0.98 |
| A. G. Ording, et al | 2013 | 0.92 | 0.74 | 1.10 |

**(6) Dataset of all-cause dementia and breast cancer survivors received chemotherapy (No chemotherapy controls)**

| **Author** | **Year** | **HR/OR** | **LL** | **UL** |
| --- | --- | --- | --- | --- |
| A. G. Ording, et al | 2013 | 0.93 | 0.23 | 3.8 |
| X. L. Du, et al | 2010 | 0.9 | 0.59 | 1.39 |
| N. N. Baxter, et al | 2009 | 0.7 | 0.55 | 0.93 |

**(6) Dataset of subgroup analysis**

| **Author** | **Year** | **HR/OR** | **LL** | **UL** |
| --- | --- | --- | --- | --- |
| ***SERMs &*** ***dementia*** | | | | |
| Chao Cai, et al | 2024 | 0.89 | 0.81 | 0.96 |
| M. R. Thompson, et al | 2021 | 1.18 | 1.03 | 1.36 |
| G. L. Branigan, et al | 2020 | 0.84 | 0.8 | 0.88 |
| L. M. Sun, et al | 2016 | 0.83 | 0.69 | 0.98 |
| A. G. Ording, et al | 2013 | 0.92 | 0.74 | 1.10 |
| ***AIs & dementia*** | | | | |
| Chao Cai, et al | 2024 | 0.93 | 0.88 | 0.99 |
| M. R. Thompson, et al | 2021 | 0.94 | 0.86 | 1.03 |
| G. L. Branigan, et al | 2020 | 0.83 | 0.76 | 0.89 |
| ***ET & AD*** | | | | |
| Chao Cai, et al | 2024 | 0.93 | 0.88 | 0.98 |
| M. R. Thompson, et al | 2021 | 0.99 | 0.87 | 1.15 |
| G. L. Branigan, et al | 2020 | 0.82 | 0.75 | 0.9 |
| A. G. Ording, et al | 2013 | 0.51 | 0.22 | 1.20 |
| ***ET & VaD*** | | | | |
| M. R. Thompson, et al | 2021 | 0.93 | 0.74 | 1.16 |
| A. G. Ording, et al | 2013 | 1.20 | 0.82 | 1.70 |

# Supplementary Figures


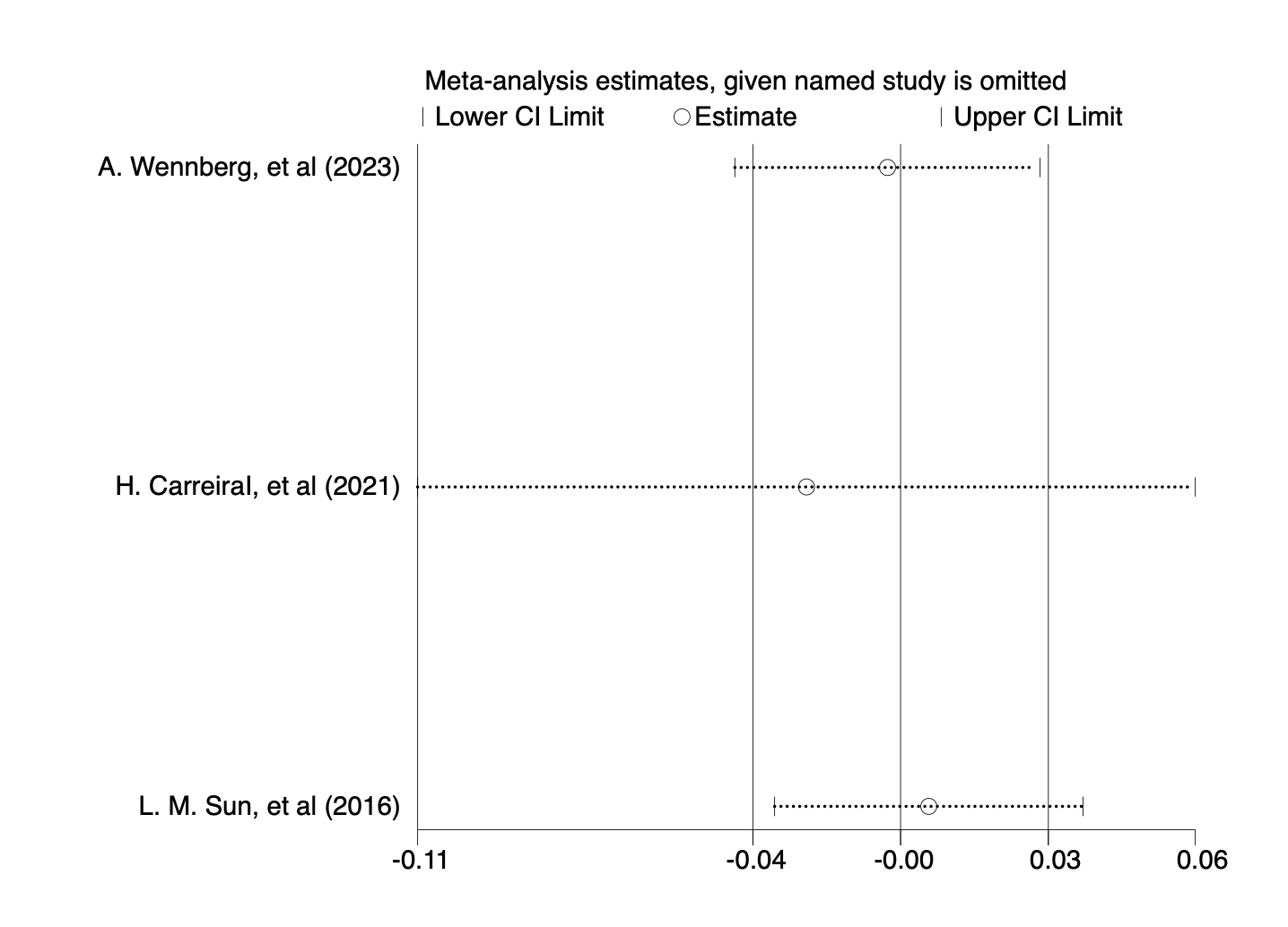


**Supplementary Figure 1.** Sensitivity analysis of the association of breast cancer survivor and all-cause dementia.


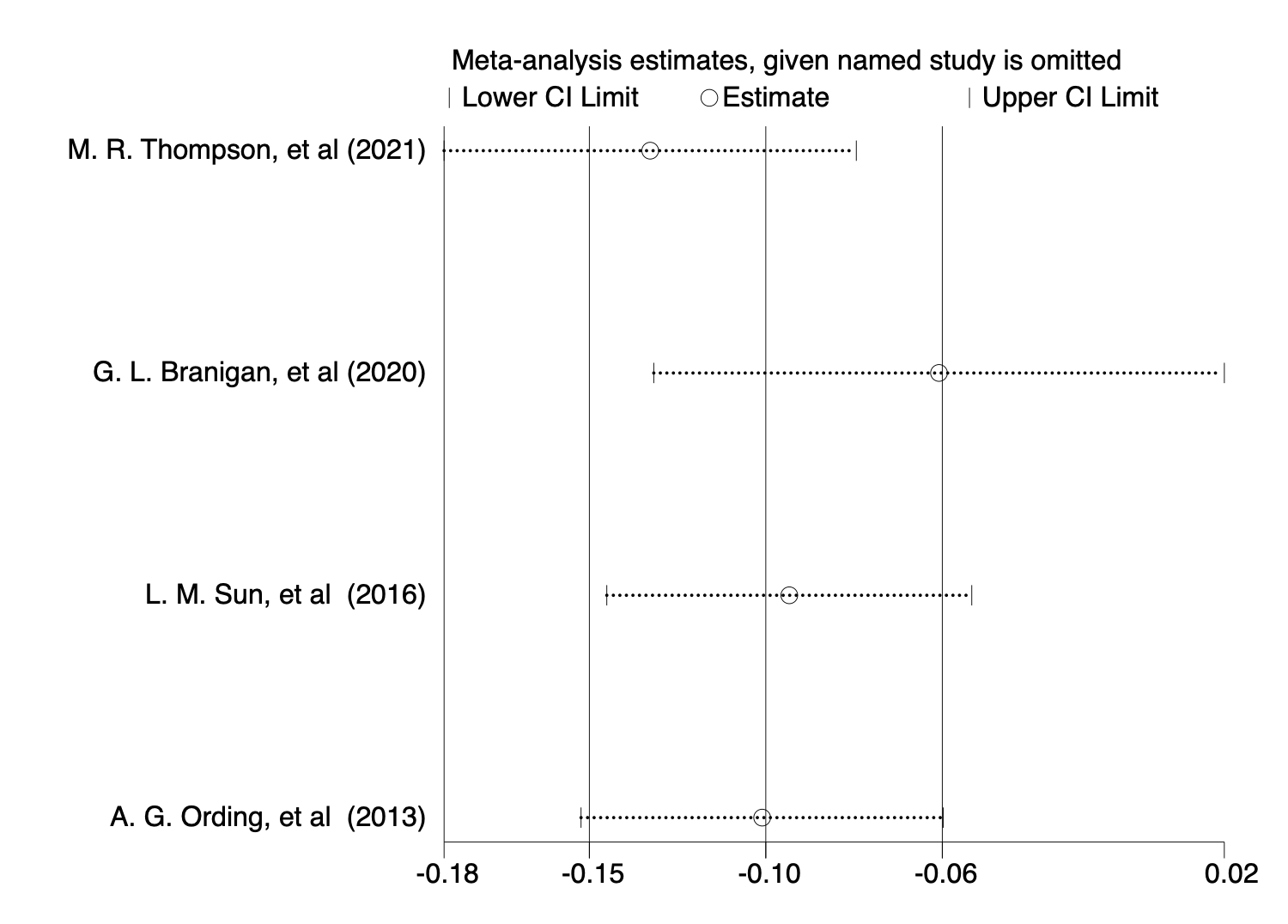


**Supplementary Figure 2.** Sensitivity analysis of the association of breast cancer survivor received ET and all-cause dementia.


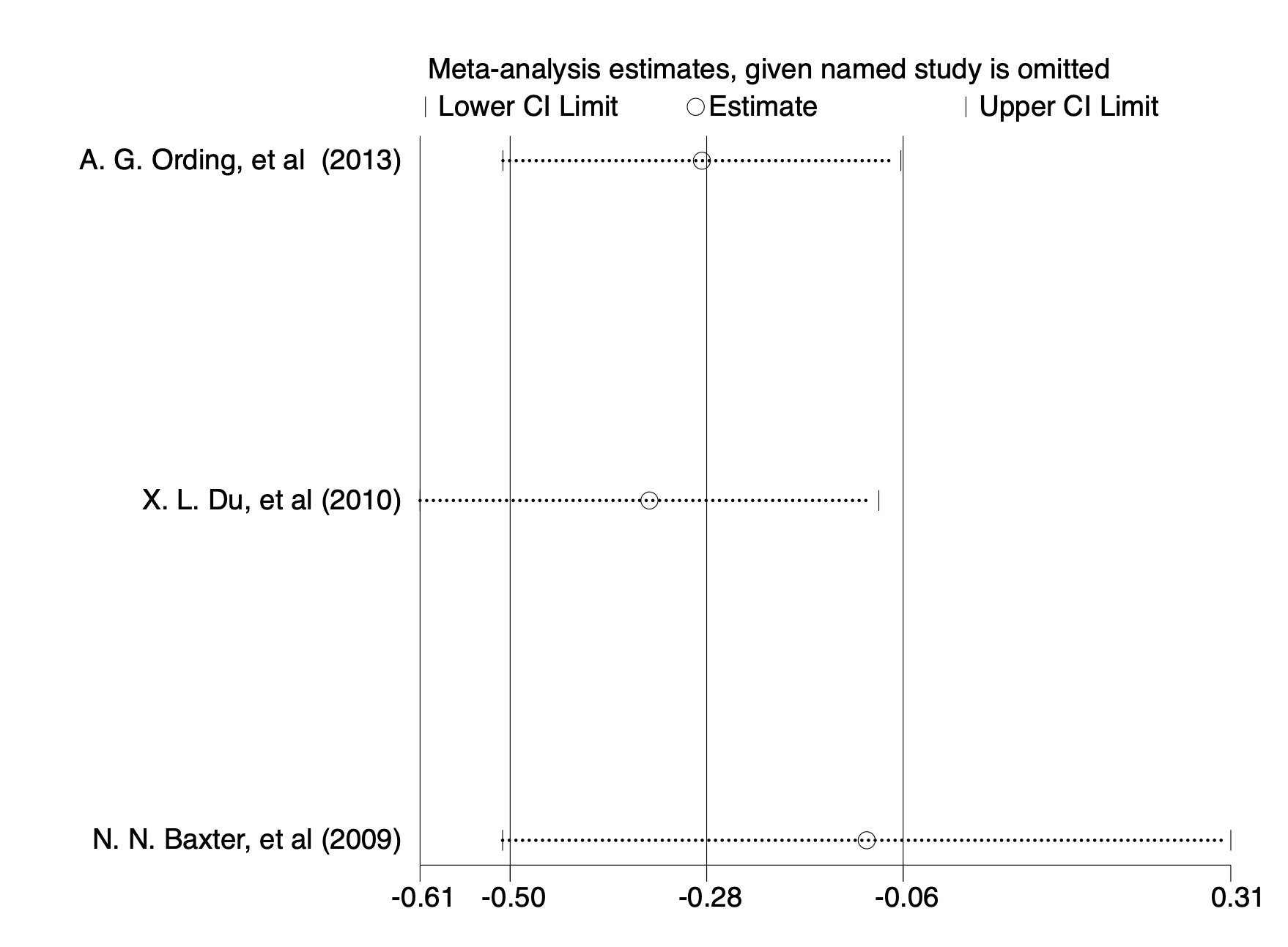


**Supplementary Figure 3.** Sensitivity analysis of the association of breast cancer survivor received chemotherapy and all-cause dementia.


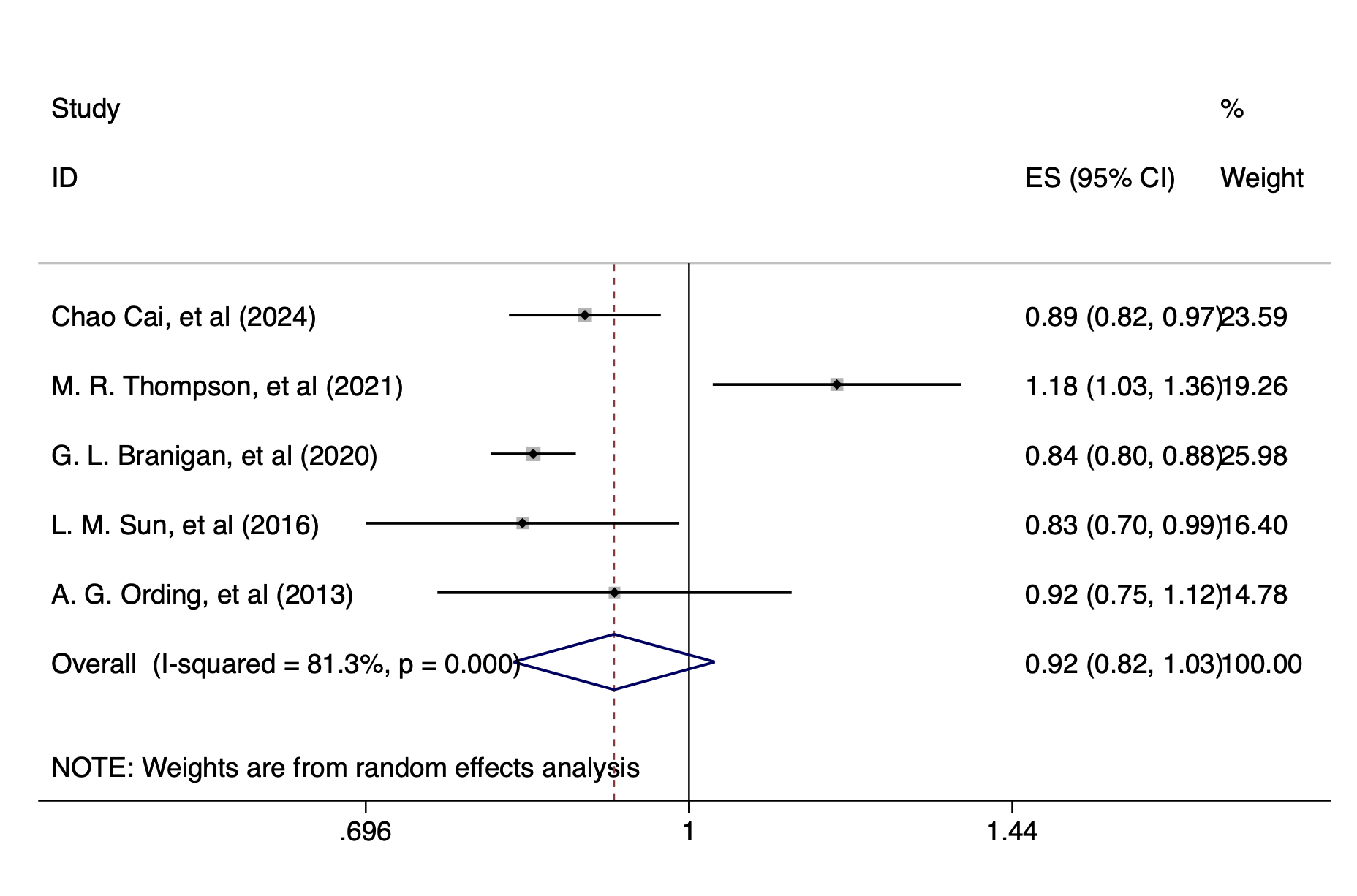


**Supplementary Figure 4.** Forest plot for the risk of dementia in breast cancer survivor undergoing SERMs and dementia. ES, effect size.

**
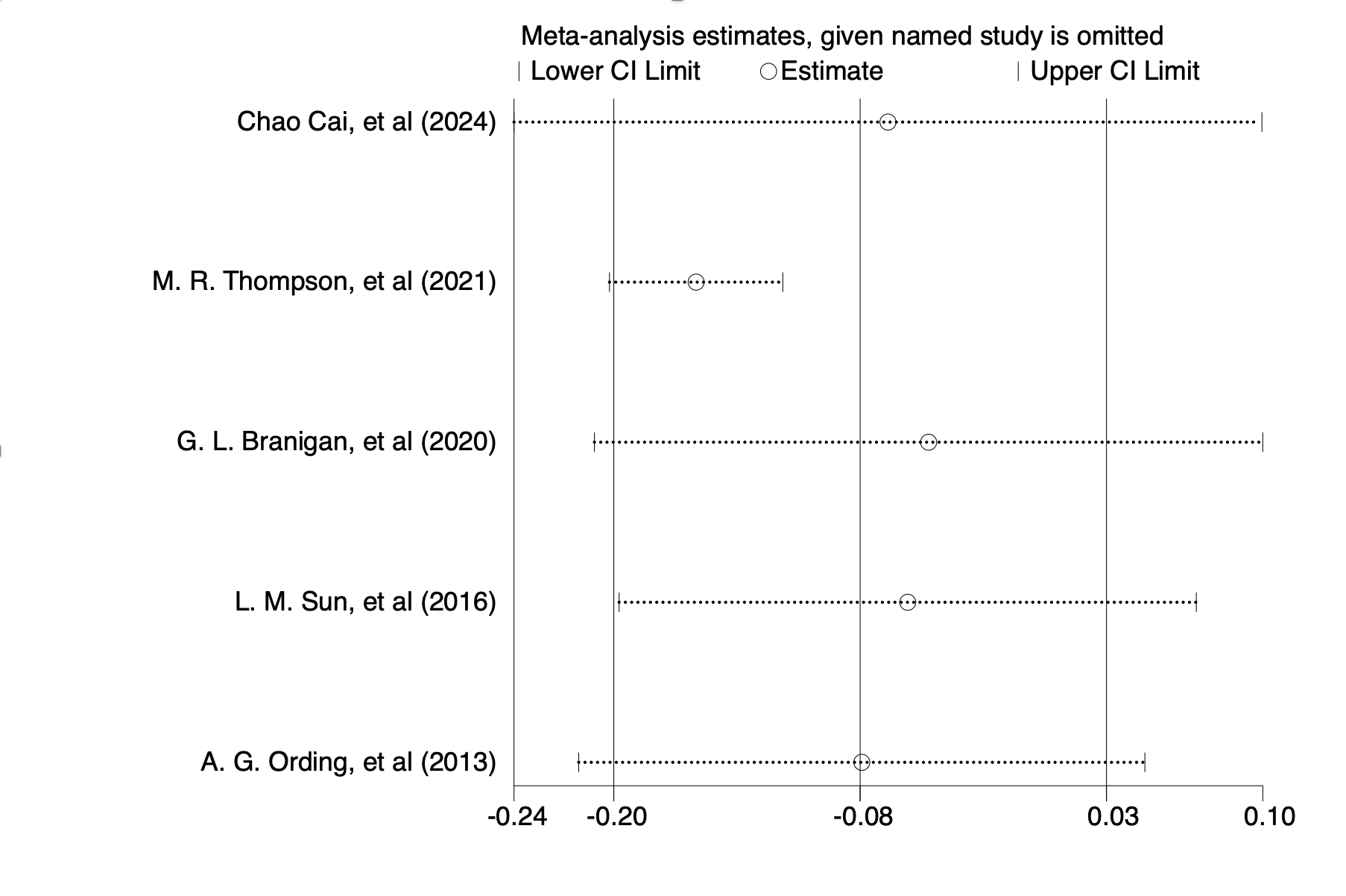
**

**Supplementary Figure 5.** Sensitivity analysis of the association of breast cancer survivor undergoing SERMs and dementia.


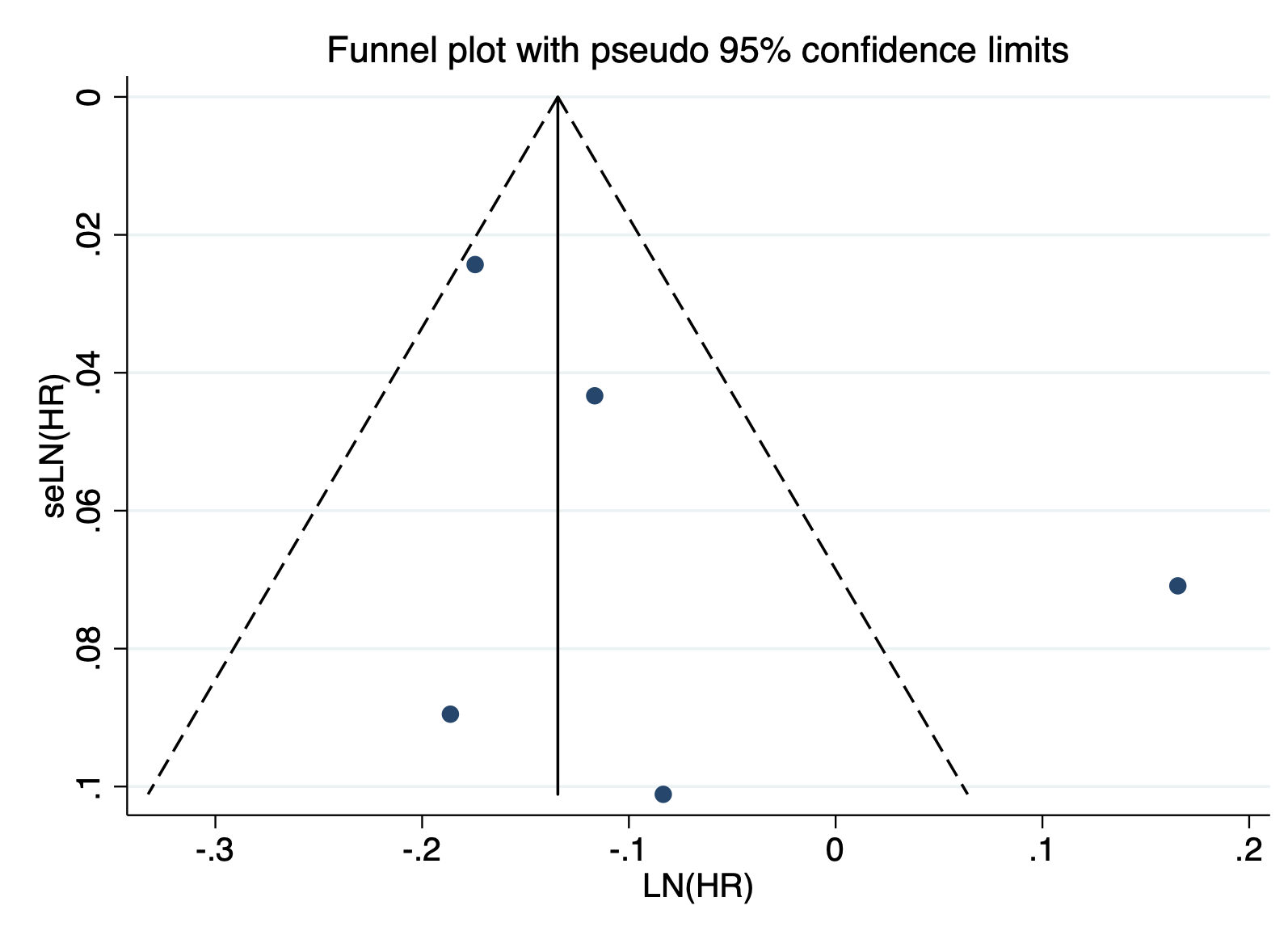


**Supplementary Figure 6.** Funnel plot for dementia in breast cancer survivor undergoing SERMs.

**
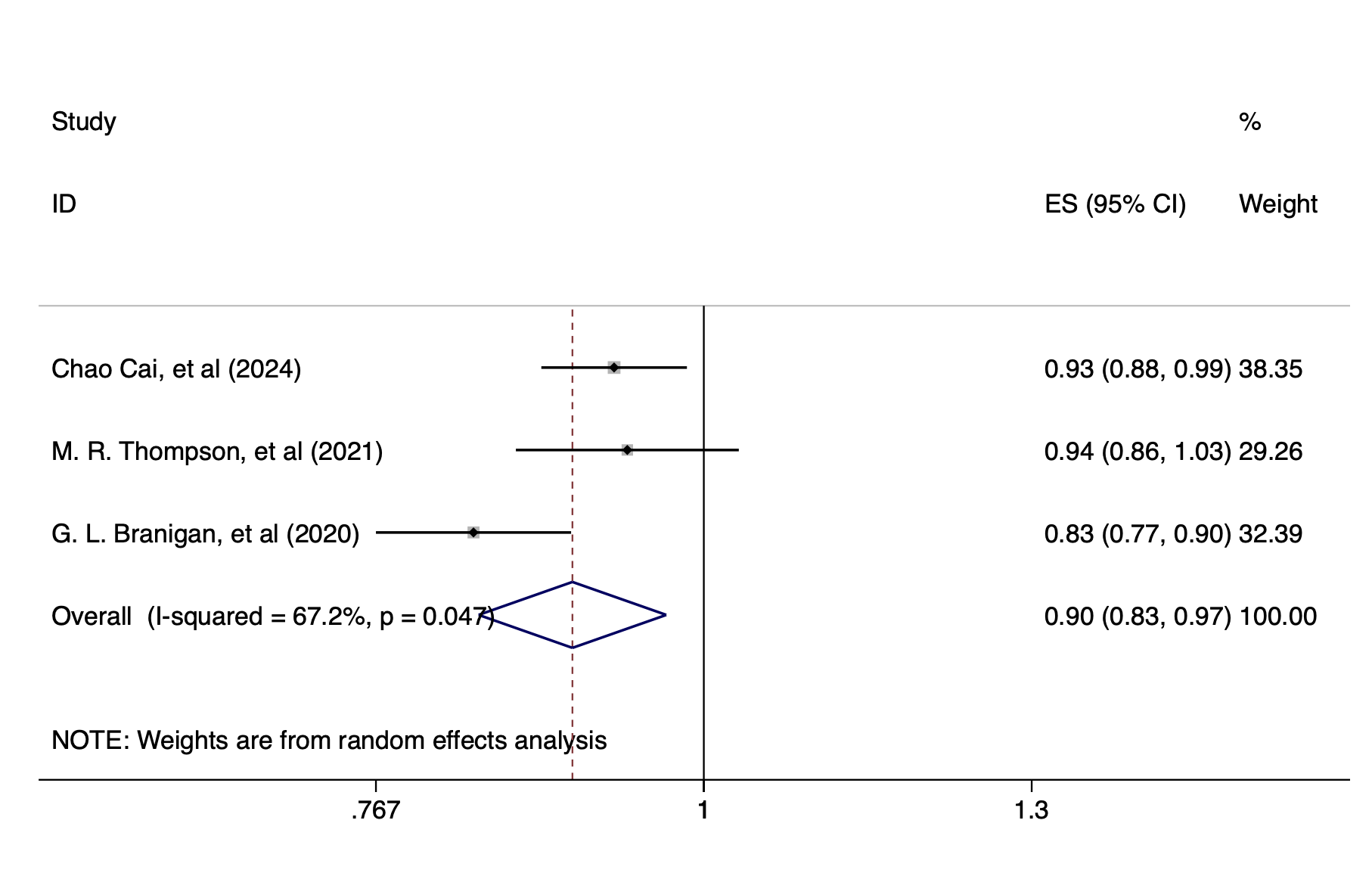
**

**Supplementary Figure 7.** Forest plot for the risk of dementia in breast cancer survivor undergoing AIs and dementia. ES, effect size.

**
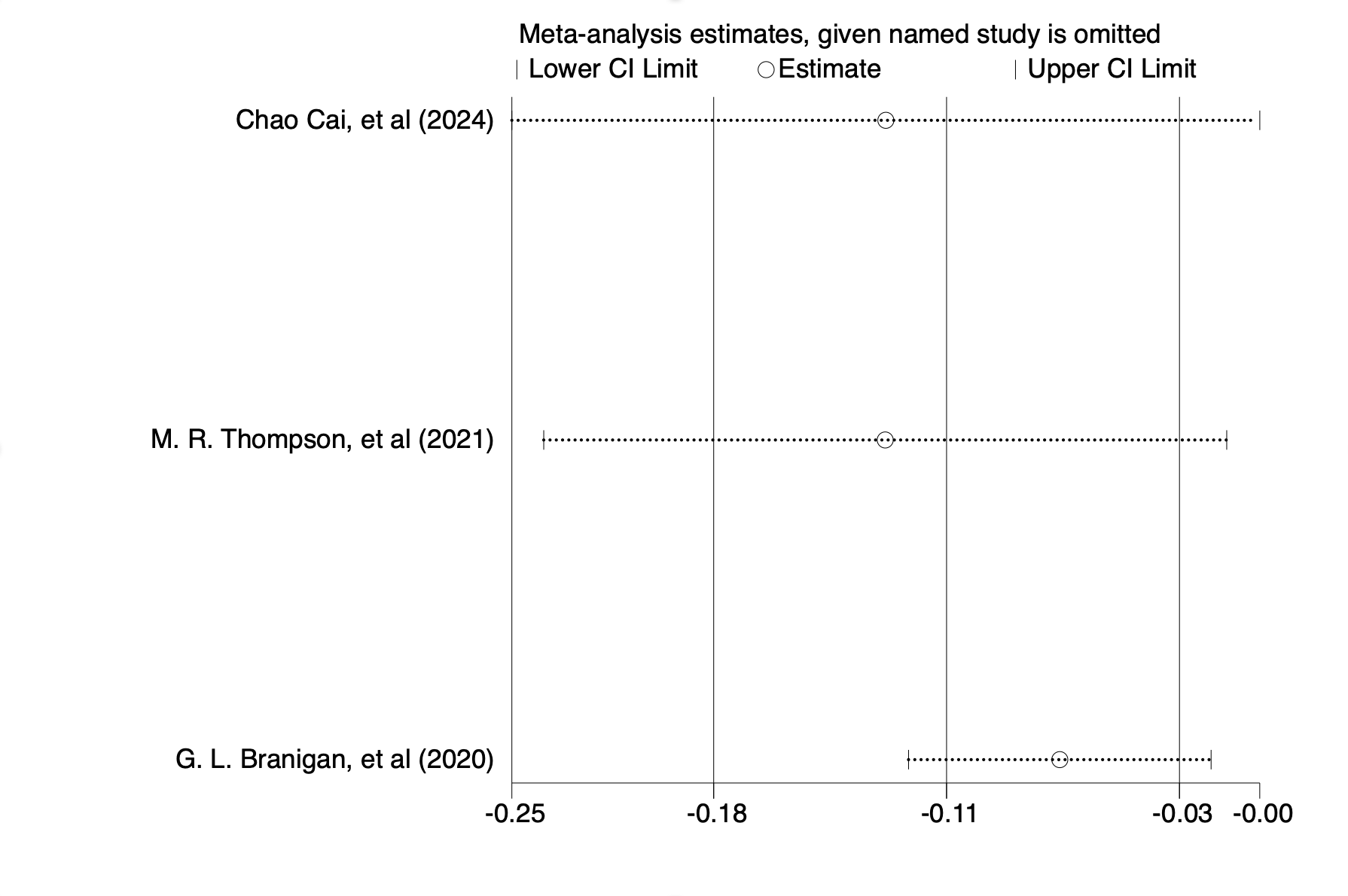
**

**Supplementary Figure 8.** Sensitivity analysis of the association of breast cancer survivor undergoing AIs and dementia.

**
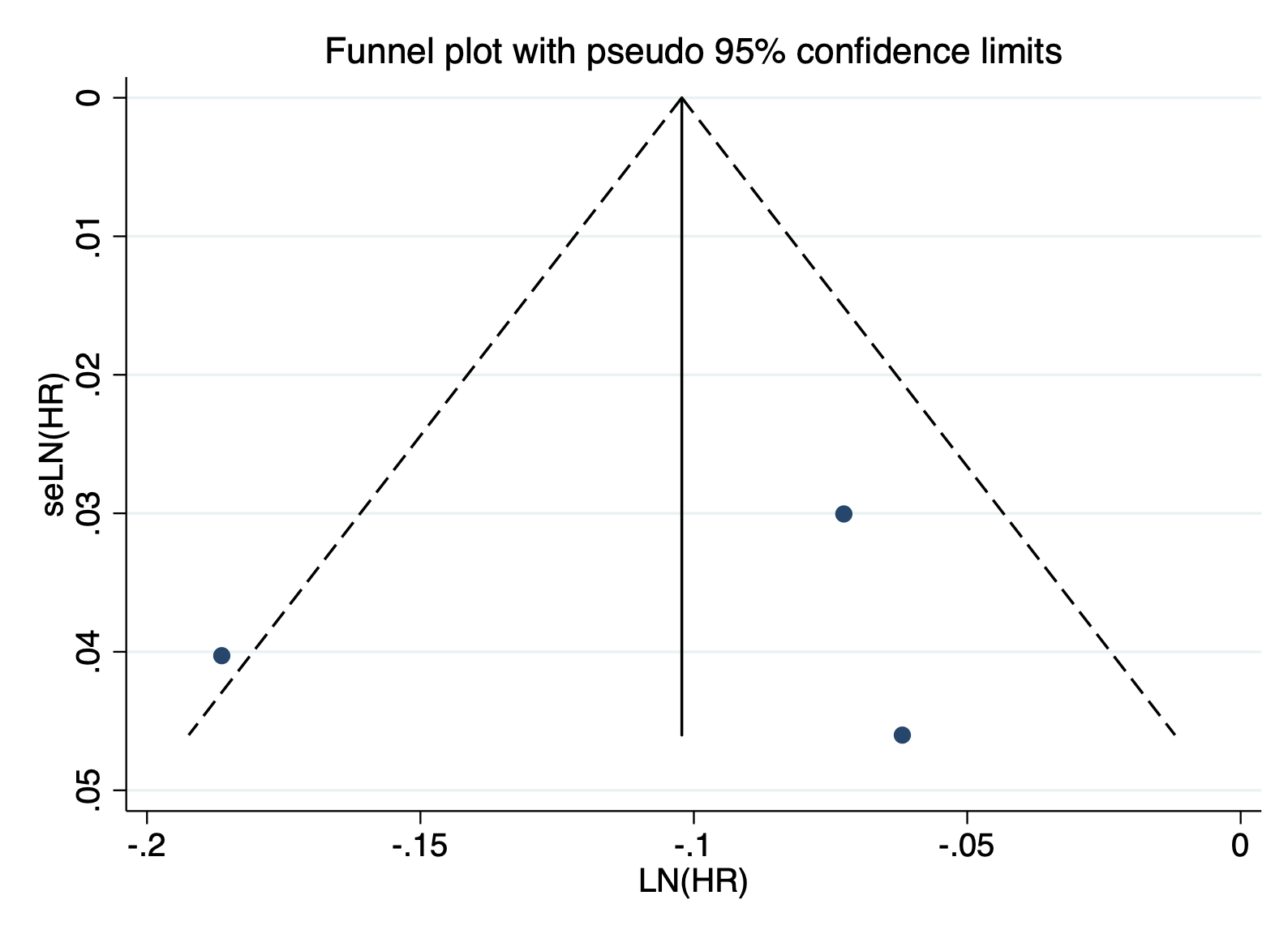
**

**Supplementary Figure 9.** Funnel plot for dementia in breast cancer survivor undergoing AIs.

**
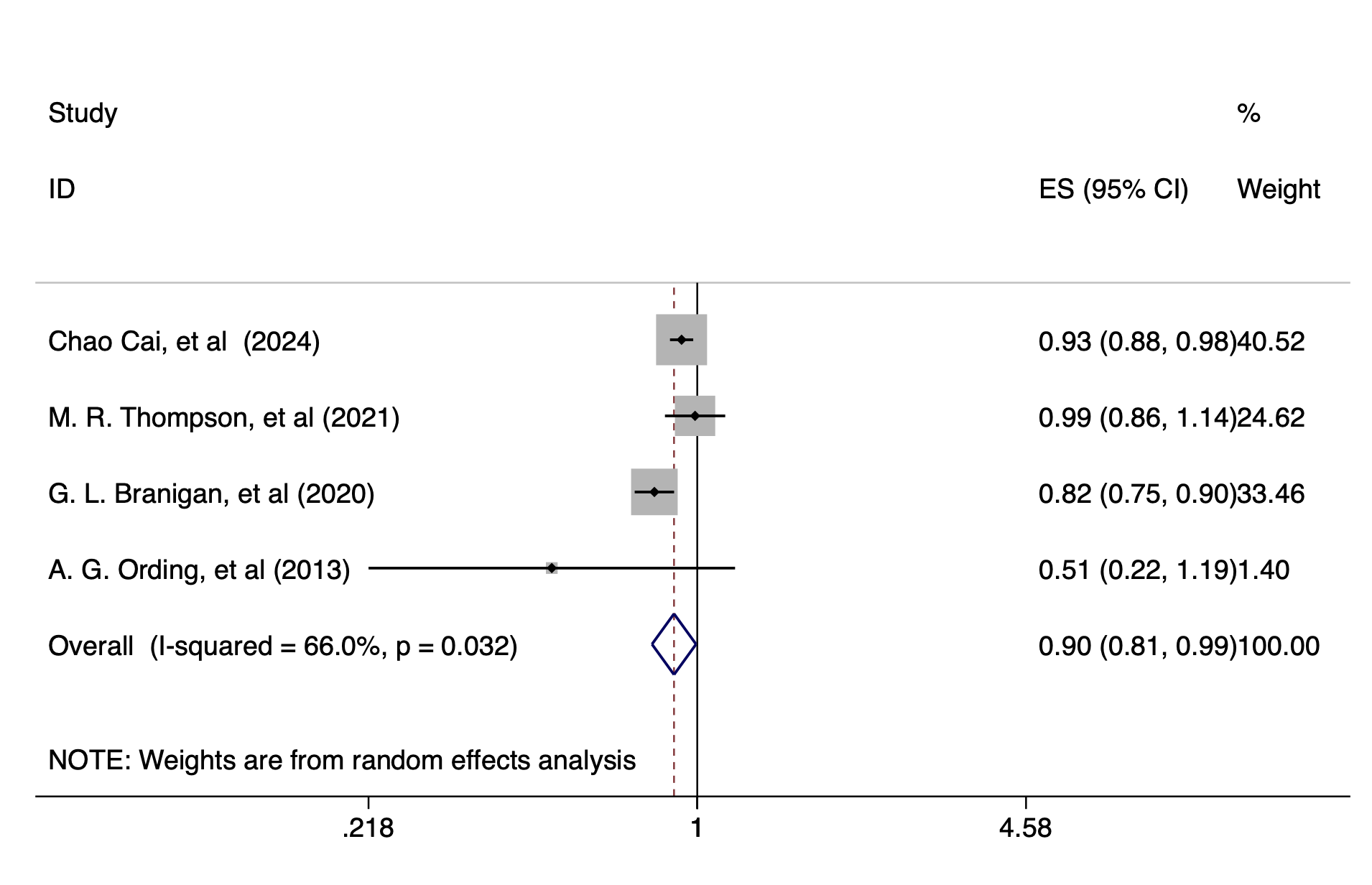
**

**Supplementary Figure 10.** Forest plot for the risk of dementia in breast cancer survivor received ET and AD. ES, effect size.

**
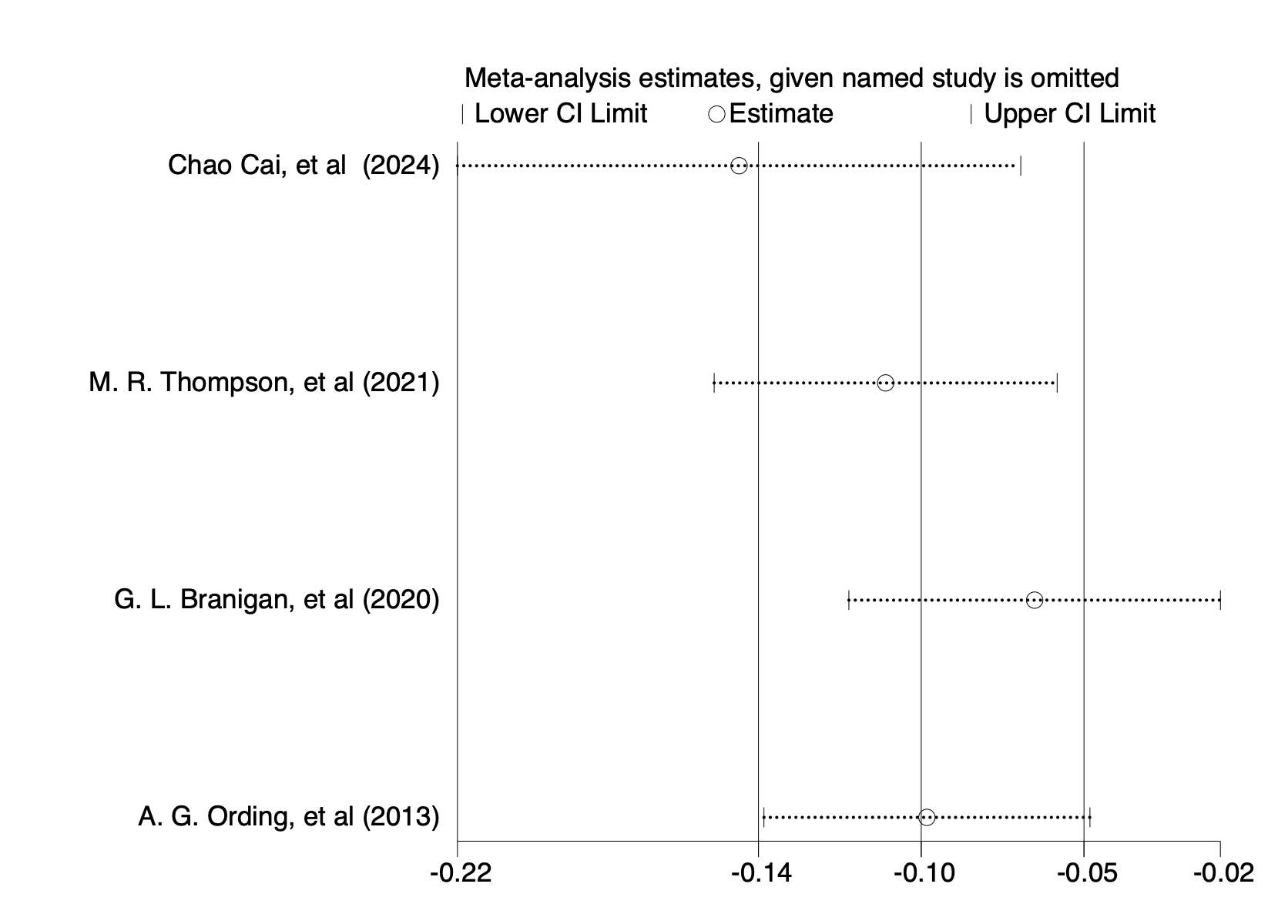
**

**Supplementary Figure 11.** Sensitivity analysis of association for the risk of dementia in breast cancer survivor received ET and AD.

**
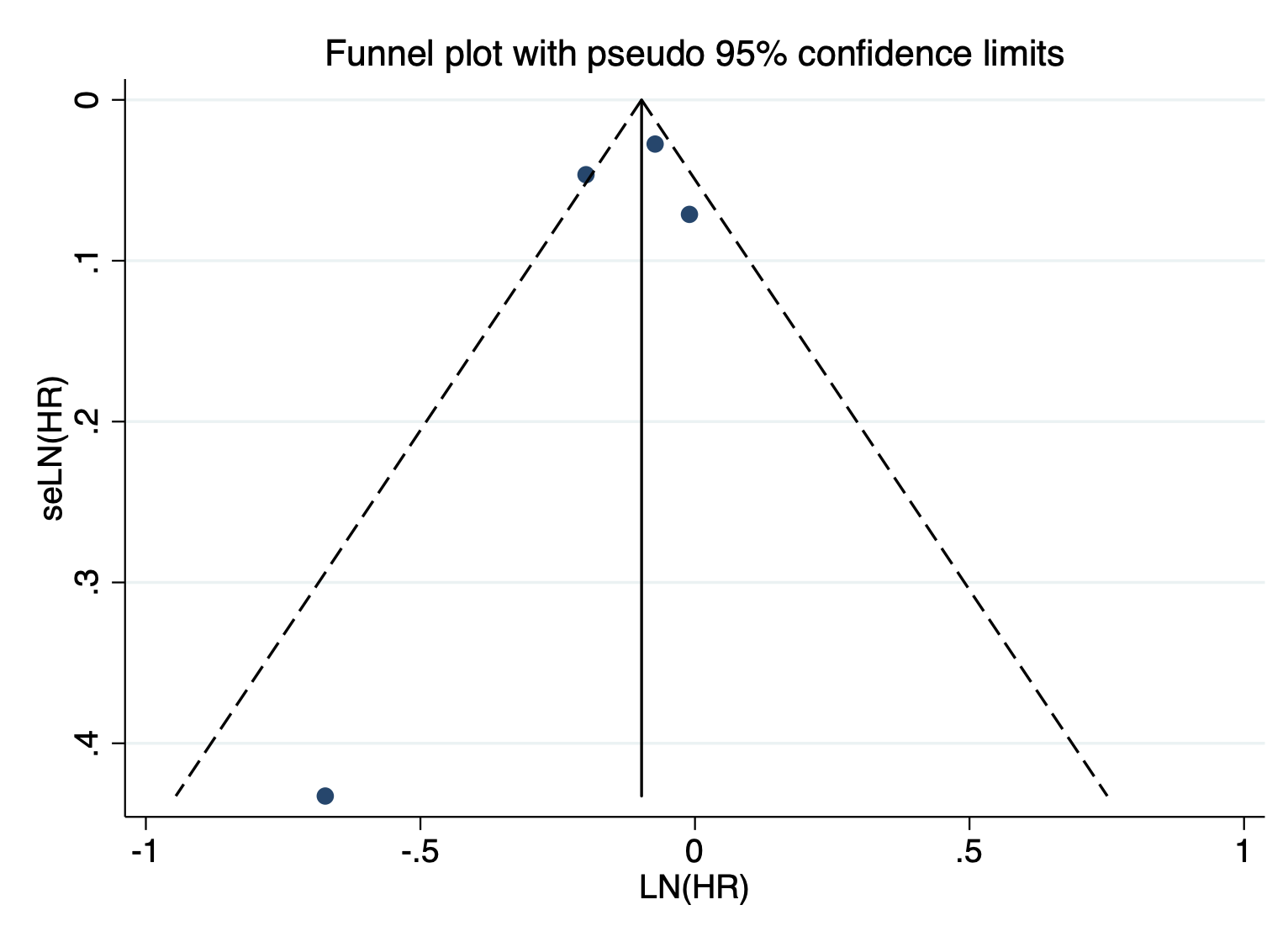
**

**Supplementary Figure 12.** Funnel plot for dementia in breast cancer survivor received ET and AD.


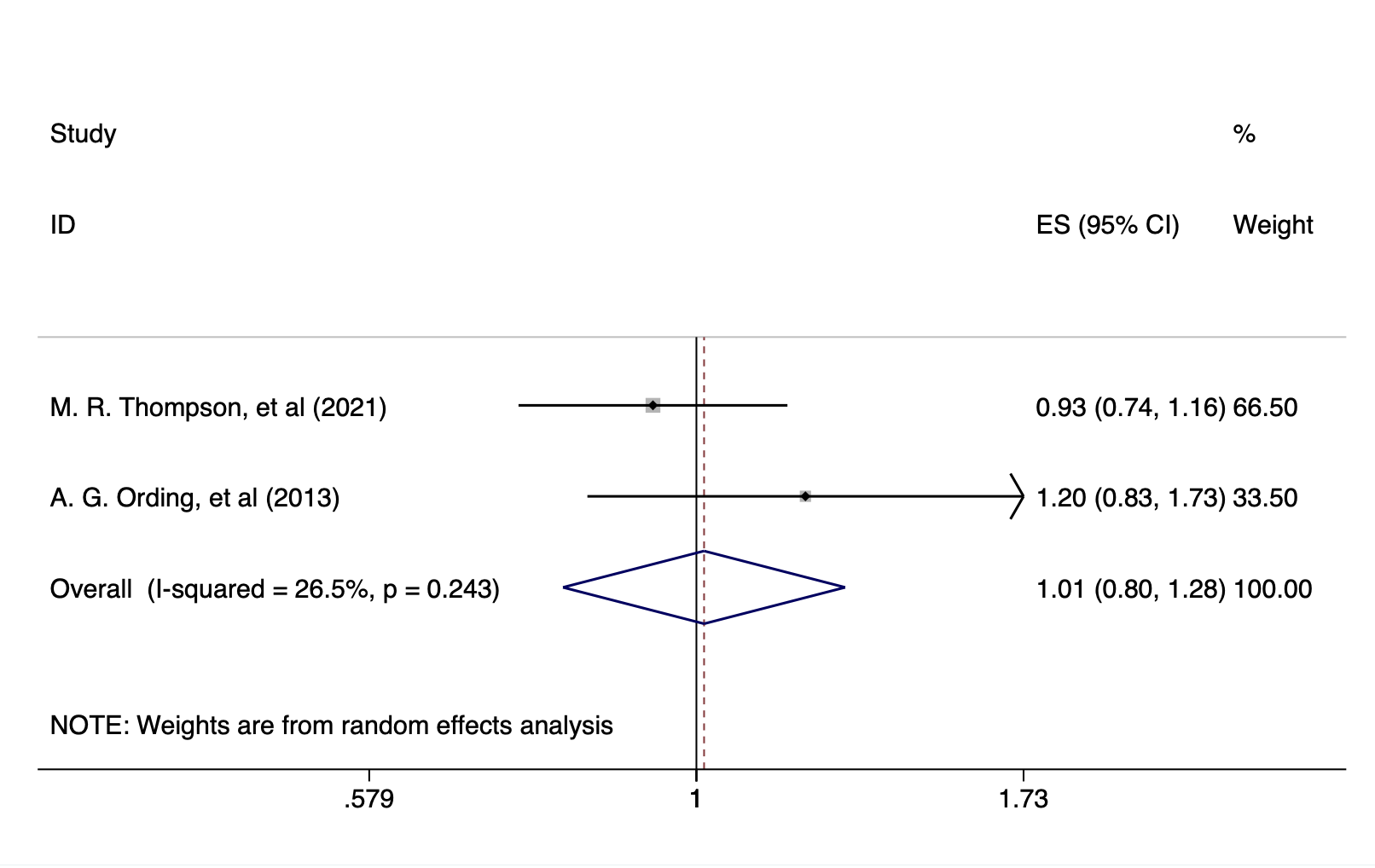


**Supplementary Figure 13.** Forest plot for the risk of dementia in breast cancer survivor received ET and VaD. ES, effect size.
